# Male Breast Cancer: Clinicopathological, Immunohistochemical and Radiological Study

**DOI:** 10.5146/tjpath.2020.01490

**Published:** 2020-09-15

**Authors:** Bermal Hasbay, Filiz Aka Bolat, Hüseyin Özgür Aytaç, Murat Kuş, Ayşin Pourbagher

**Affiliations:** Department of Pathology, Başkent University, Faculty of Medicine, Adana, Turkey; Department of General Surgery, Başkent University, Faculty of Medicine, Adana, Turkey; Department of Radiology, Başkent University, Faculty of Medicine, Adana, Turkey

**Keywords:** Male breast cancer, Diagnosis, Survival

## Abstract

***Objective:*** To evaluate the pathological and radiological features, immunohistochemical profile and treatment methods of primary male breast carcinoma cases diagnosed at our center.

***Material and Method:*** The pathology archive between 2006 and 2019 was reviewed and the data of 27 male patients diagnosed as primary breast cancer were retrospectively evaluated.

***Results:*** The age of the patients ranged between 40-86 years. The left breast was involved in 17 patients. The mean tumor diameter was 2.35 ± 1.09 cm. Of the 27 cases, 8 were dead and 19 were alive. The mean follow-up duration was 37.45 ± 24.84 months. The mean estimated life expectancy was 65±14.7 months. The most common complaint was a swelling in the breast. The time interval between the onset of complaints and admittance to hospital ranged from three months to two years. The most common histopathological diagnosis was invasive carcinoma - no special type. The most common surgical procedure was mastectomy with lymph node dissection. Nine patients had metastatic lymph nodes. In terms of the hormone profiles, 24 were Estrogen receptor positive, 21 were Progesterone receptor positive and six were Her2/neu positive. Three patients had triple-negative tumors.

***Conclusion:*** Male breast carcinoma is a rare disease but its frequency has been increasing recently. As breast cancer is more commonly attributed to women, the diagnosis is usually delayed until later stages in males. Public awareness should therefore be increased and breast cancer should be considered in the differential diagnosis especially in the presence of breast swelling and complaints related to the breast skin so that the appropriate biopsy can be obtained without delay.

## INTRODUCTION

Male breast cancer (MBC) is a very rare disease accounting for nearly 1% of all cancers in males (1–7). However, its incidence has recently been increasing with approximately 2000 to 2500 new cases reported annually in the United States (1,3,8–12). Although male breast cancer occurs in all age groups, it is frequently observed between 60 and 70 years of age on average (2,3,10,12–14). Genetic factors, BRCA2 mutations, family history, obesity, Klinefelter’s syndrome, gynecomastia, liver disease, orchitis, undescended testicle, alcohol use, exogenous estrogen and testosterone use, and radiation are accused in the etiology (1,8,9,15–17). Patients mostly present with a painless mass, nipple discharge, skin ulceration, or nipple retraction (2,4). The most common type is invasive carcinoma - no specific type (IC-NST) (2,3,9). Ultrasonography (USG) and Magnetic Resonance Imaging (MRI) are used radiologically, and biopsy and/or surgical excision is required for definitive diagnosis (2,5). The aim of this study was to evaluate the rare male breast cancer in terms of clinical, pathological, radiological, and therapeutic methods and to discuss the results with the literature.

## MATERIALS and METHODS

We retrospectively evaluated the pathology archive of our hospital between January 2006 and August 2019 and included 27 cases diagnosed as primary breast cancers in this study. Clinical follow-up of the cases was obtained from the electronic data system and the record archive of our hospital.

A 12-year electronic data search was performed using the laboratory information system with the ‘breast’ and ‘male’ keywords in the diagnostic line. Biopsies had been obtained from a total of 87 patients, including 38 with tumors, 36 gynecomastia cases, and 7 lipomas, 2 hamartomas, one granulomatous inflamation, one fibroadenoma + gynecomastia, one cystic lymphangioma, and one ductal ectasia. Eight of these 38 tumor cases were metastatic to the breast and 30 were primary cases. One of the 30 primary tumor cases was a liposarcoma of the breast and two were pure ductal carcinoma in situ (DCIS) without invasive components. These three cases were excluded and a total of 27 patients were included in the study (Table 1). The patients were evaluated retrospectively for age, tumor size, tumor localization, histological grade; hormone profile with estrogen receptor (ER), progesterone receptor (PR), Her2/neu; the American Joint Committee on Cancer (AJCC) Tumor, Node, Metastases (TNM) stage; progression, recurrence, survival, radiological features, surgery, and therapy modalities (adjuvant, neoadjuvant chemotherapy and/or radiotherapy and hormonotherapy).

**Table 1:** Pathological results of biopsy in 87 male patients.

**Status**	**Diagnosis**	**Number of patients (%)**
**Malignant**		38 (43.7)
	IC-NST	23 (26.5)
	Metastatic tumor	8 (9.3)
	Invasive lobular carcinoma	2 (2.3)
	Ductal carcinoma in situ	2 (2.3)
	IMPC with apocrine features	1 (1.1)
	Liposarcoma	1 (1.1)
	Mixed carcinoma (IC-NST+Cribriform carcinoma)	1 (1.1)
**Benign**		49 (56.3)
	Gynecomastia Lipoma Hamartoma Granulomatous mastitis Fibroadenoma+gynecomastia Ductal ectasia Cystic Lymphangioma	36 (41.5) 7 (8.1) 2 (2.3) 1 (1.1) 1 (1.1) 1 (1.1) 1 (1.1)
		** 87 (100%)**

Immunohistochemical (IHC) assays were performed using monoclonal antibodies against ER (Clone EP1, Code M3643, Dako, Denmark), PR (Clone Y85, 60-0056-7, Genemed, Germany), and Her 2/neu (Code A0485, Dako, Denmark). ER and PR status was studied by obtaining positive and negative control tissues and using ready-to- use solutions in the Leica bond max device. We followed the ASCO and CAP recommendations for reporting the IHC assay results for ER, PR and Her2/neu. All cases with at least 1% positive cells were considered receptor positive for ER and PR (18).

Her2/neu status can be determined by assessing protein expression on the membrane of tumor cells using IHC or by assessing the number of Her2/neu gene copies using in situ hybridization (ISH). The results for Her2/neu testing by IHC were reported according to the intensity and the percentage of positive staining in tumor cells (0, 1+, 2+, 3+). Scores of 0 and 1+ were considered negative for Her2 amplification. A score of 3+ was considered positive. A score of 2 was considered equivocal and ISH was ordered for confirmation (19).

Statistical analysis was performed using the SPSS statistical package software (Version 17.0, SPSS Inc., Chicago, IL, USA). All numerical data were expressed as median values (minimum-maximum) or as proportions. The association with overall survival was analyzed using the log-rank test to examine their relationship when different variables were applied. The survival curve was plotted using standard Kaplan-Meier methodology.

Written consent was not obtained from the patients since the study was designed retrospectively and needed no consent.

## RESULTS

The age of the patients ranged from 40 to 86 years (mean age: 62.52 years, median age: 61 years). The left breast was involved in 17 of 27 (63%) patients and the right breast in 10 of 27 (37%). The mean tumor diameter was 2.35 ± 1.09 cm (min. 0.6 cm, max. 4.5 cm). Of the 27 cases, 8 (29.6%) were dead, 19 (70.4%) were alive. The mean follow up time was 37.45 ± 24.84 months (4-80 months). The estimated life expectancy of all patients was 65 ± 14.7 months. Most of the patients presented with swelling of the breast, but to a lesser extent they suffered from areolar wounds, redness, and bloody discharge.

**Figure 1:**
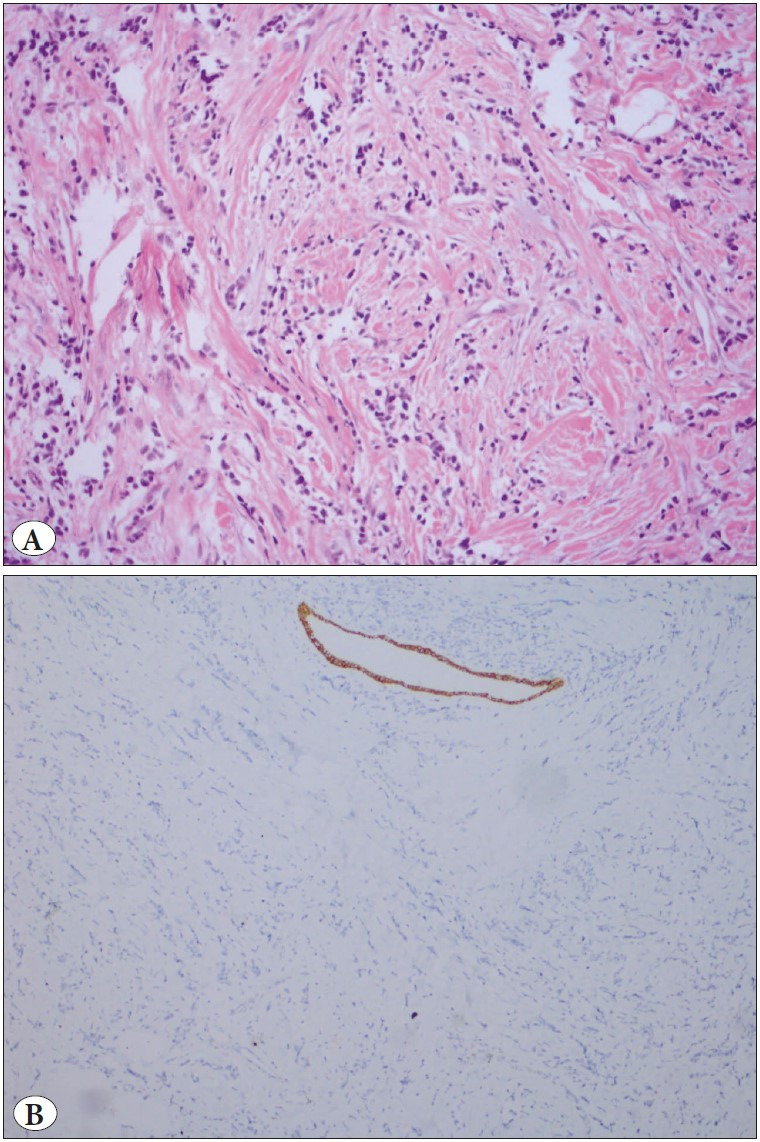
**A)** Invasive lobular carcinoma. Discohesive cells in desmoplastic stroma (H&E; x200). **B)** Loss of E-cadherin expression is typical of lobular carcinoma cells (IHC; x100).

**Figure 2:**
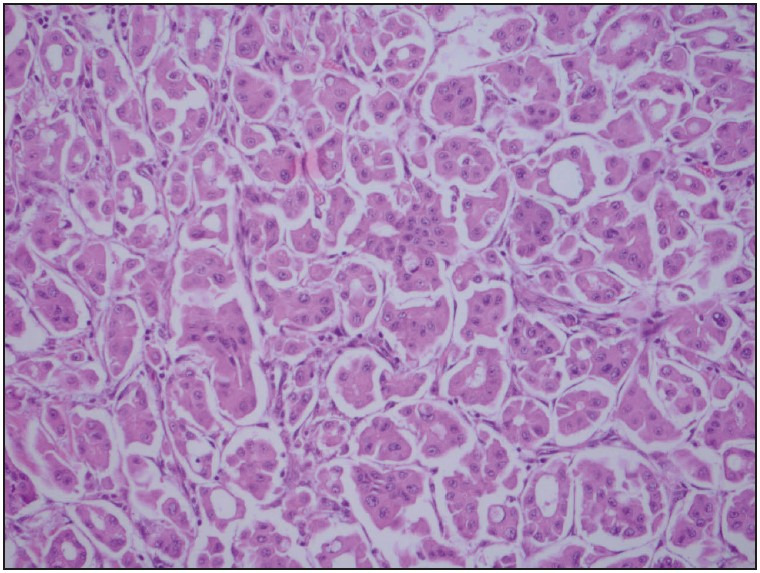
Invasive micropapillary carcinoma with apocrine features tumor cells with granular, eosinophilic cytoplasm and enlarged nuclei with prominent nucleoli (H&E; x200).

The most common histopathological diagnosis was IC-NST (85.2%). There were two (7.4%) invasive lobular carcinoma cases (ILC-Figure 1), both with negative epithelial-cadherin staining (Figure 1); one case (3.7%) of invasive micropapillary carcinoma with apocrine features (IMPC-Figure 2), and one case of mixed carcinoma (IC-NST + Cribriform). Three of the cases with IC-NST contained neuroendocrine differentiation areas. According to the Modified Bloom and Richardson score, 14 cases were grade 3 (51.9%), 11 cases were grade 2 (40.7%), and two cases were grade 1 (7.4%).

When evaluated in terms of pT, 7 cases (25.9%) were pT1, 11 cases (40.8%) were pT2, and 2 cases (7.4%) were pT4. Two of the 7 (25.9%) cases with missing pT were diagnosed with ready-to-use paraffin blocks and five with core biopsies and were no longer followed-up. Two patients underwent excisional biopsy, 3 patients underwent mastectomy, 11 patients underwent mastectomy with lymph node dissection (LND), and 4 patients underwent mastectomy with sentinel lymph node dissection (SLND). Metastatic lymph nodes were observed in 9 out of 15 cases with lymph node sampling, whereas lymph nodes were reactive in 6 cases (summarized in Table 2).

**Table 2:** Clinico-pathological characteristics of 27 patients with male breast cancer.

**n(%)**	**n(%)**
**Age group** Mean age 62.52 [40-86] Median age 61	**Her 2-neu** Positive 6 (22.2) Negative 21 (77.8)
**Tumor location** Right 10 (37) Left 17 (63)	**PN** pN0 6 (22.2) pN1 5 (18.6) pN2 3 (11.1) pN3 1 (3.7) pNx 12 (44.4)
**Tumor size** Mean size 2.35 cm [0.6-4.5] Median size 2 cm	**Tumor subtype** IC-NST 23 (85.2) ILC 2 (7.4) IMPC with apocrine features 1 (3.7) Mixed (IC-NST + Cribriform) 1 (3.7)
**pT** pT1 7 (25.9) pT2 11 (40.8) pT4 2 (7.4) Unknown 7 (25.9)	**Metastasis** Bone 2 (7.4) Pleura 1 (3.7) Bone + Liver 1 (3.7) No metastasis 23 (85.2)
**Nuclear grade** G1 2 (7.4) G2 11 (40.7) G3 14 (51.9)	**Surgery** Mastectomy 3 (11.1) Mastectomy and ALND 11 (40.8) Mastectomy and SLND 4 (14.8) Excisional biopsy 2 (7.4) Unknown 7 (25.9)
**Estrogen receptor (ER)** Positive 24 (88.9) Negative 3 (11.1)	**Systemic therapy** CT + RT 3 (11.1) CT+RT+TTZ 1 (3.7) CT+RT+ TMX 7 (25.9) CT+RT+TMX+TTZ 4 (14.8) TMX 2 (7.4) RT +TMX 1 (3.7) KT +TMX+TTZ 1 (3.7) No follow up patient 8 (29.7)
**Progesterone receptor (PR)** Positive 21 (77.8) Negative 6 (22.2)	**Final status** Alive 19 (70.4) Ex 8 (29.6)

**SLND:** Sentinel lymph node dissection, **CT:** Chemotherapy, **RT:** Radiotherapy, **ALND:** Axillary lymph node dissection, **pT:** Pathologic tumor stage, **pN:** Pathologic nodal stage, **IC-NST:** Invasive carcinoma - Carcinoma of no special type, **TTZ:** Trastuzumab , **TMX:** Tamoxifen.

**Figure 3:**
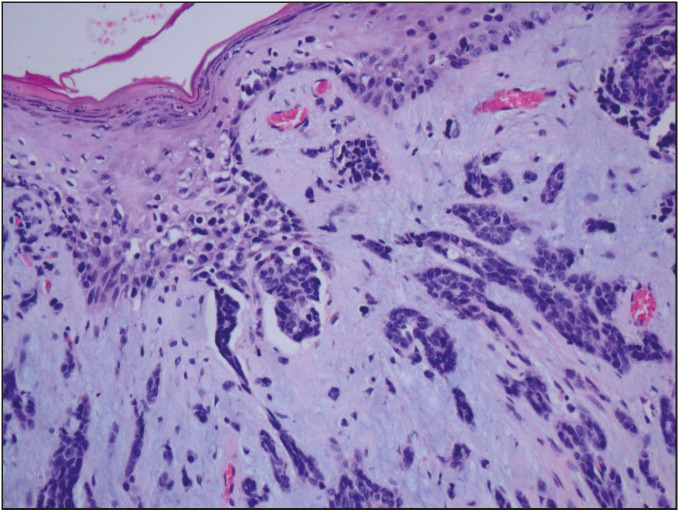
Nipple epidermis containing Paget cells with palestained cytoplasm and irregular nuclei (H&E; x200).

When examined radiologically, 20/27 of the cases had a USG. Four patients had mammography. On USG, 19/20 cases had an irregularly confined lesion with a solid lobule appearance that was suspicious in terms of malignancy. In another case, the USG appearance was reported to be compatible with bilateral lipoma but the biopsy was reported as IMPC with apocrine features. Four of our patients had breast carcinoma as well as a second primary carcinoma consisting of one small cell lung carcinoma, one lung adenocarcinoma, one prostate adenocarcinoma, and one thyroid papillary carcinoma. Four of our cases metastasized: two to the bone, one to the pleura, and the other to the bone and liver. Paget’s disease was present in three cases (Figure 3). The hormone profile was positive for ER in 24 (88.9%) and negative in three (11.1%). PR was positive in 21 (77.8%) patients and negative in six (22.2%) patients. Her2/neu was negative in 21 (77.8%) and positive in six (22.2%) cases. There were 3 (11.1%) patients with triple-negative breast cancer. DCIS accompanied the main pathology in 10 (37%) of the patients. Two (7.4%) patients had multifocal tumors, which were ILC and IMPC with apocrine features. Of the 27 cases, 19 (70.4%) were being followed-up and 8 (29.6%) were out of follow-up. Five cases did not come back after core biopsy, and two cases had a ready-made paraffin block for diagnosis confirmation, and one case was lost to follow-up after 20 months. Thus eight cases could not be followed-up.

Six of the patients who died had IC-NST, one had IC-NST with cribriform carcinoma, and the other had ILC.

The treatment protocols of 19 patients were chemotherapy (CT) + radiotherapy (RT) in 15 and Tamoxifen (TMX) and/or Trastuzumab (TTZ) were added to the treatment in case of hormone receptor positivity. Two of the other four patients were treated only with TMX, one with RT + TMX, and one with CT + TMX + TTZ.

## DISCUSSION

Although male breast cancer is very rare compared to women, its incidence has been increasing in recent years. Approximately 2000-2500 new cases are added each year in the United States, while this rate is 1/100000 in India (1,7–9,11,20). We should therefore pay attention to the clinical, genetic and epidemiological features of male breast cancer. Although seen in all age groups, it is mostly observed between the ages of 60 and 65 years (1,3,4). In our series, the ages ranged from 40 to 86 years, with an average age of 61 years.

The most common complaint is generally a palpable mass in the breast. Although signs such as nipple discharge, mass, contraction, scarring, and redness in the breast are seen in many cases, they are generally ignored and delays in diagnosis are therefore experienced as the hospital admissions are late (13). In our series, the most common complaint was swelling of the breast, followed by a non-healing wound on the nipple, bloody discharge, and redness. There were delays that ranged from 3 months to 2 years between the onset of the complaints and first symptom and admission to the hospital. The reason for the delay may be related to the fact that breast cancer is more commonly associated with women in the community. The diagnosis is made by the history, physical examination, radiological methods, and histopathological examinations. We first use USG radiologically at our institution in the event of a suspected abnormality during a clinical examination. Due to the rarity of male breast cancer, we attempt to avoid likely unnecessary radiation. If sonographic findings are suspicious, a biopsy is the next step. Male BC is diagnosed by mammography and/or USG and confirmed by a core biopsy that is always performed following a suspicious clinical examination.

Male breast cancer is mostly seen in the left breast (2). It was also more commonly observed in the left breast in our series (17/27). Age, race, family history, obesity, genetic factors (especially BRCA2 mutations), gynecomastia, Klinefelter syndrome, Cowden syndrome, liver diseases that cause an estrogen increase, cirrhosis, ionized radiation and prolonged heat exposure (increases prolactin level) due to environmental factors, alcohol, and excess consumption of red meat are mostly accused in the etiology (1,2,7–9,12,13,17). In our series, two patients had a history of radiation (due to lung cancer), three patients had gynecomastia, one patient had obesity, three patients had diabetes and CRF (dialysis patient), and four patients had a history of breast carcinoma in their sisters. One of our patients with a family history also had an alcohol use disorder and diabetes, and another had gynecomastia. Gynecomastia can be observed in 6-38% of men with breast cancer (16). Three of our patients (11.1%) had gynecomastia.

Although the mean tumor size reported in the literature is 2 to 3.5 cm, it can vary between 0.5 and 12.5 cm (2,3,13). In our series, tumor size ranged from 0.6 to 4.5 cm, with an average of 2.35 cm, in accordance with the literature.

Among male breast cancers, IC-NST (80-90%) is the most common histopathological diagnosis followed by papillary carcinomas. Less often, ILC, mucinous carcinoma and apocrine carcinoma are detected (8,14). In our series, 23 IC-NST, two ILC, one IMPC with apocrine features, and one mixed carcinoma (IC-NST with cribriform carcinoma) were observed, consistent with the literature. A total of two ILC were identified in our study, both with negative epithelial-cadherin staining. Paget’s disease is a rare disease and constitutes 1% of breast cancers. It is an eczematous skin disease, usually associated with an underlying breast cancer (6). Considering that male breast cancers constitute 1% of all breast cancers, the incidence of Paget’s disease in male breast cancer is very low. In our series, Paget’s disease was associated with breast cancer in three (11.1%) cases (Figure 3). Therefore, Paget’s disease should be considered in the differential diagnosis of non-healing wounds of the nipple, and biopsy should be performed to exclude an underlying carcinoma.

In terms of grade, an MBC study found that 73% were grade 3 while another retrospective study of 1180 MBC from the SEER database demonstrated that 39% were grade 3 (20,21). In our study, 14 cases were grade 3 (51.9%) and 11 cases were grade 2 (40.7%). In a few studies, the median survival has been shown to be significantly poor in high-grade (grade 3) tumors (14). However, no such significant correlation was seen between tumor grade and outcome (p= 0.65) in our study.

Axillary involvement is present in approximately 30-50% of the cases at the time of diagnosis (2,14) and was present in 9/27 (33%) of our cases. Most male breast cancers are usually positive for ER (65-97%) and PR (60-85%) (2,4,13,14,22,23). The Her2/neu positivity rate is 3-28% (14,22,24). The rate of triple-negative breast carcinoma varies between 3 and 19% (14,24). In our series, ER was positive in 24 (88.9%), PR was positive in 21 (77.8%), and Her2 / neu was positive in 6 (22.2%) patients. FISH was performed in patients with a Her2/neu score of 2 by immunohistochemistry to confirm the diagnosis.**

**Prostate, lung, skin, gastrointestinal system, and thyroid cancers can be seen as a secondary malignancy in 5-33% of male breast cancer patients (2,3,8,23). In our series, the secondary malignancies were two lung cancers, one prostate cancer, and one thyroid cancer.

Since male breast cancer is rare, standard approaches have historically relied on the results of trials in female breast cancer (22). Treatment methods vary according to the tumor stage and surgery, CT, RT, and hormone treatment methods can be used in single or combined forms (3,17). Surgically, mastectomy rather than breast-conserving surgery was performed in the vast majority of cases in the literature (12,17). Mastectomy was also the most common procedure in our series.

Our study has the limitations of a retrospective study from a single institution conducted over a long period. On the other hand, we believe the fact that all of our patients underwent a multidisciplinary diagnosis and follow-up process contributed to data standardization.

Since the prognosis of male breast cancer is the same as that of female breast cancer of the same stage, early diagnosis is the most important factor for treatment success.

For this reason, breast cancer should be considered in the differential diagnosis and an appropriate biopsy should be performed in case of complaints about breast skin and breast swelling in order to diagnose the condition at an earlier stage. It is important to raise public awareness by explaining that breast cancer is not unique to women, to teach self breast examination to men, and even to start breast screening programs similar to those for women for early diagnosis.

## Conflict of Interest

No conflict of interest was declared by the authors.

## FUNDING

The authors declared that this study has received no financial support.
